# Cloning and Functional Verification of Endogenous *U6* Promoters for the Establishment of Efficient CRISPR/Cas9-Based Genome Editing in Castor (*Ricinus communis*)

**DOI:** 10.3390/genes14071327

**Published:** 2023-06-23

**Authors:** Masatake Kanai, Kazumi Hikino, Shoji Mano

**Affiliations:** 1 Laboratory of Organelle Regulation, National Institute for Basic Biology, Okazaki 444-8585, Japan; 2 Basic Biology Program, Graduate Institute for Advanced Studies, The Graduate University for Advanced Studies, SOKENDAI, Okazaki 444-8585, Japan

**Keywords:** *Ricinus communis*, *U6* promoter, particle delivery system

## Abstract

Castor (*Ricinus communis*) seeds are rich in a type of hydroxy fatty acid called ricinoleic acid, which is in high demand for the production of plant-based plastics, lubricants, and hydraulic oils. However, the high content of ricin, a toxic protein, in these seeds has restricted further expansion in the area of castor cultivation. Therefore, the development of ricin-free castor is needed. Genome editing technology, although successfully applied in several plant species, is still in the developing stages in castor and awaits the identification of an endogenous *U6* promoter with robust function. Here, we searched for *U6* small nuclear RNA (snRNA) genes in the castor genome. This led to the identification of six *U6* snRNA genes. The promoters of these *U6* snRNA genes were cloned, and their function was examined in castor cells using the particle delivery method. The results showed that a U6 promoter length of approximately 300 bp from the transcription start site was sufficient to activate gene expression. This study provides insights into the endogenous castor *U6* promoter sequences and outlines a method for verifying the function of *U6* promoters in plants using the particle delivery system.

## 1. Introduction

Castor (*Ricinus communis*) is an herbaceous plant of the Euphorbiaceae family that accumulates high amounts of hydroxy fatty acids, particularly ricinoleic acid, in its seeds [1]. Castor oil is composed of approximately 90% ricinoleic acid and is used as a lubricant and hydraulic oil owing to its high fluidity over a wide temperature range [1,2,3]. In addition, castor oil is almost the only feedstock for polyamide 11, a representative plant-derived engineered plastic of high commercial value [1,2,3,4]. Because of these properties, castor oil is in high demand, which continues to grow annually.

Castor has long been cultivated on a large scale in India, China, and Brazil as a non-food commercial crop and is increasingly being cultivated on poor soils in Mozambique, Ethiopia, and other countries [5]. In addition, because of its high heat and drought tolerance, castor can be grown on land unsuitable for other crops [6,7]. However, castor seeds contain ricin, an enzymatic protein that is highly toxic to humans and livestock because of its ability to inactivate eukaryotic ribosomes [8]. The presence of ricin in seeds is one of the main factors that limits the cultivation of castor, and the development of ricin-free castor genotypes is therefore needed.

CRISPR/Cas9, a genome editing technology, is the most promising method for producing ricin-free castor [9]. The application of the CRISPR/Cas9 system relies on (1) the availability of genome sequence information for accurate guide RNA (gRNA) design and (2) an established transformation method. The genome sequence of castor was released as a draft in 2010 [10], and chromosome-level genome assembly information was published in 2022 [11]. Although transformation methods for castor were recently reported [12], more suitable technologies for genome editing in castor are being established.

One of the problems with genome editing in plants is low editing efficiency [13]. Methods to improve editing efficiency have been extensively studied. Kor and colleagues recently summarized that gRNAs need to be vigorously and constantly expressed to improve genome editing efficiency, indicating that the promoter sequence used to express gRNAs has a significant impact on editing efficiency [14]. Furthermore, the authors reported that the promoter region of the *U6* small nuclear (snRNA) gene was ideal for gRNA expression in various organisms [14]. Additional studies have shown that the use of endogenous *U6* snRNA promoter sequences (henceforth *U6* promoter) is necessary to achieve high editing efficiency [15,16,17,18]. In this study, we report the cloning of six *U6* promoters from the castor genome and demonstrate their ability to express a transgene in castor cells. The development of a castor-optimized CRISPR/Cas9 system is essential for efficient genome editing in castor.

## 2. Materials and Methods

### 2.1. Plant Material and Growth Conditions

To induce germination, castor seeds were placed on a wet paper towel and incubated for 72 h at 22 °C in the dark. Three-day-old seedlings were transferred to 1/5000a Wagner pots filled with cultured soil and grown under long-day conditions (16 h light/8 h dark) at 25 °C in a growth chamber (NK System, Osaka, Japan). One-tenth strength of Hoagland solution (1 L) was supplied to the plants once every 3 days. Experimental studies on plants, including the collection of plant material, were conducted in accordance with relevant institutional, national, and international guidelines and legislation.

### 2.2. Plasmid Construction

The *RcU6* promoter fragments, with *att*B4 and *att*B1R sequences at their 5′ and 3′ ends, respectively, were amplified by PCR with specific primer sets (Table S1) and cloned into the pDONR P4-P1R entry vector (Thermo Fisher Scientific, Tokyo, Japan) using the Gateway BP recombination method (Thermo Fisher Scientific, Tokyo, Japan). The *Venus-PTS1* sequence, with *att*B1 and *att*B2 sequences at its 5′ and 3′ ends, respectively, was amplified by PCR with specific primer sets (Table S1) and cloned into the pDONR221 entry vector (Thermo Fisher Scientific, Tokyo, Japan) using the Gateway BP recombination method (Thermo Fisher Scientific, Tokyo, Japan). To generate various *RcU6* promoter constructs, *Venus-PTS1* and the DNA fragments cloned into pDONR P4-P1R or pDONR221 were transferred into the R4pGWB401 destination vector [19] using the Gateway LR recombination reaction.

### 2.3. Transient Expression with the Particle Delivery System

A total of 10 mg of gold particles (1.0 μm diameter) coated with the plasmid (20 mg) were introduced into 25-day-old first or second castor true leaf cells using a Helios Gene Gun (Bio-Rad, Hercules, CA, USA), according to the manufacturer’s instructions. During the bombardment, castor leaves were placed on wet filter paper, and helium gas was injected at a pressure of 200 psi. The bombarded samples were incubated in the dark at 22 °C for 16 h.

### 2.4. Confocal Microscopy

Castor leaf cells injected with the plasmid-coated gold particles were observed under a confocal laser scanning microscope (LSM510META; Carl Zeiss, Jena, Germany) [20]. Emission filters BP535-590 were used to detect signals from Venus-PTS1.

### 2.5. Nucleotide Acession Numbers

The nucleotide sequences of the six *U6* promoters in this study are registered in DDBJ/GenBank/EMBL under accession numbers LC765373 for *U6-1*, LC765374 for *U6-2*, LC765375 for *U6-3*, LC765376 for *U6-4*, LC765377 for *U6-5*, and LC765378 for *U6-6*.

## 3. Results and Discussion

### 3.1. Castor U6 snRNA Gene Identification and Promoter Cloning

Endogenous promoters driving gRNA expression are essential for improving genome editing efficiency [15,16,17,18]. The upstream regions of endogenous *U6* snRNA genes are most commonly used as promoters for genome editing [14]. The transcribed regions of *U6* snRNA genes are highly conserved among species [21]. Therefore, *U6* promoters have been searched for in various plant species and identified in their genomes based on the transcribed sequence of *U6* snRNA genes in *Arabidopsis thaliana*. However, *U6* snRNA genes reportedly exist as multicopies in many organisms and also as pseudogenes [22,23]. Therefore, functional verification of *U6* snRNA genes is essential for identifying a functional promoter in the plant species of interest.

**Figure 1:**
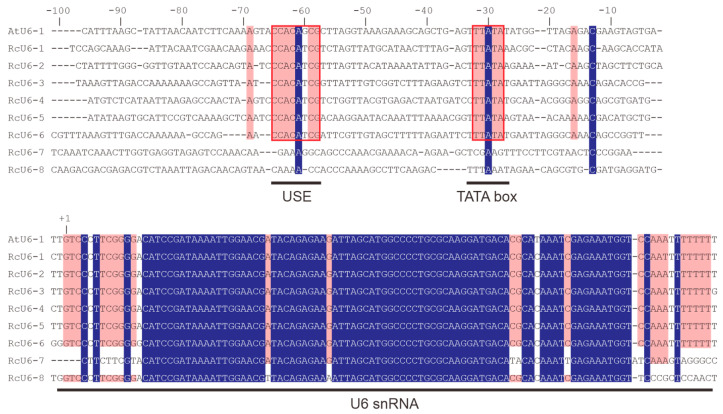
Alignment of eight castor *U6* and Arabidopsis *U6-1* genes. The sequence of the transcribed region of the *U6* genes and approximately 100 bp upstream of their transcription start sites was aligned. USE, TATA box, and *U6* snRNA sequences are underlined. The conserved USE and TATA box sequences are outlined in red. Bases shaded in purple indicate those common to all DNA sequences described. Bases shaded in pink indicate those common to *AtU6-1*, *RcU6-1*, *RcU6-2*, *RcU6-3*, *RcU6-4*, *RcU6-5* and *RcU6-6*.

Based on the draft genome sequence of castor released in 2010 [10] and its chromosome-level genome assembly reported in 2022 [11], we identified eight *U6* snRNA genes in the castor genome. These include *RcU6-5* and *RcU6-7* on chromosome 1, *RcU6-3* and *RcU6-4* on chromosome 6, *RcU6-8* on chromosome 7, *RcU6-1* and *RcU6-2* on chromosome 8, and *RcU6-6* on chromosome 10 (Table 1). All eight *U6* genes showed high sequence similarity to the transcribed region sequence of the Arabidopsis *U6* snRNA1 gene (Table 1). *U6* snRNA genes possess an RNA polymerase III (POLIII) type 3 promoter, which in plants consists of a TATA box at −28 to −30 bp and a plant snRNA gene-specific element, upstream sequence element (USE), at approximately −70 bp [14]. Sequence analysis of a 100 bp sequence upstream of each of the eight *U6* snRNA genes revealed the presence of a conserved TATA box and a USE in the promoter regions of *RcU6-1* to *RcU6-6* and that of Arabidopsis U6 snRNA1 (*AtU6-1*), but these elements were absent in the promoters of *RcU6-7* and *RcU6-8* (Figure 1). Since their promoter sequences do not contain the typical POLIII type 3 promoter elements, *RcU6-7* and *RcU6-8* could be pseudogenes and may not be effective for gRNA expression. Therefore, the promoters of six of the identified snRNA genes, *RcU6-1* to *RcU6-6*, were considered for further analysis.

### 3.2. Functional Analysis of the Rc U6 Gene Promoters

**Figure 2:**
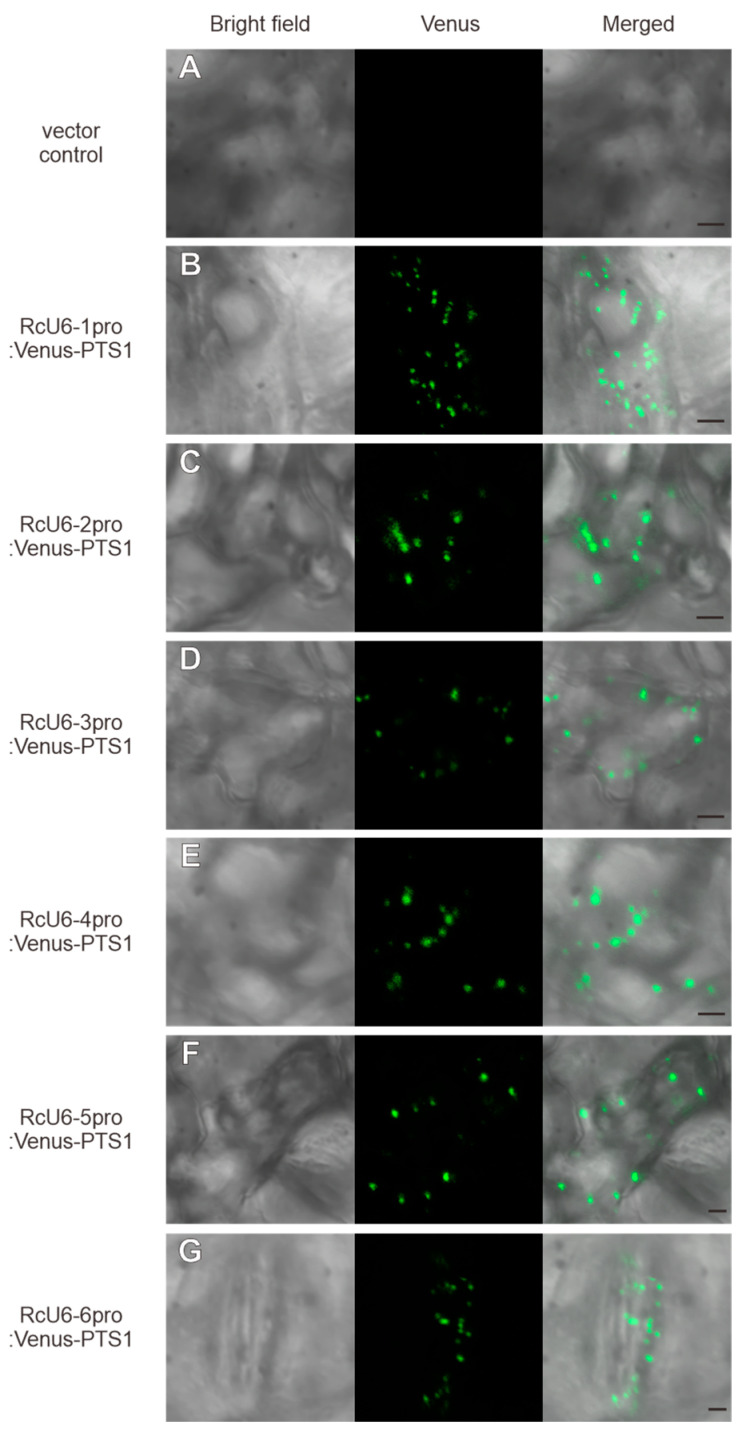
Functional verification of *RcU6* promoters. The vector without the *U6* promoter (**A**), RcU6-1pro:Venus-PTS1 (**B**), RcU6-2pro:Venus-PTS1 (**C**), RcU6-3pro:Venus-PTS1 (**D**), RcU6-4pro:Venus-PTS1 (**E**), RcU6-5pro:Venus-PTS1 (**F**), and RcU6-6pro:Venus-PTS1 (**G**) constructs were individually introduced into castor leaf cells using the particle delivery method. Scale bars in the merged pictures represent 5 µm.

Heterologous *U6* promoters have been reported to be functional in several plant species [24,25]. However, in several studies, heterologous *U6* promoters were shown to reduce genome editing efficiency compared with endogenous *U6* promoters [15,16,17,18], indicating that functional verification of promoters in native species is a better approach than testing their activity in heterologous systems. Castor has long been cultivated as a useful crop. However, the transformation of castor has been reported relatively recently [12], and a transient expression system using protoplasts for the verification of *RcU6* promoter function has not yet been established. Therefore, we carried out functional verification of the *RcU6* promoters in castor cells using the particle delivery system, which allows the transient expression of genes in a consistent manner across various plant species [26,27]. POLIII is known to be involved in the transcription of 5S rRNA, tRNAs, and small RNAs [14]. However, POLIII has also been reported to transcribe mRNAs, such as those encoding proteins [15,28]. Therefore, we performed the functional verification of *RcU6* promoters using the fluorescent protein Venus because it is easy to investigate U6 promoter activity as a fluorescent signal. The peroxisomal targeting signal 1, PTS1 [29], was ligated to the C-terminus of Venus [30] to facilitate the observation of Venus as a peroxisome-localized fluorescent signal. The *Venus-PTS1* fusion and each of the six *RcU6* promoters were further cloned into the R4pGWB401 plasmid [19,31] to generate the RcU6-1pro:Venus-PTS1, RcU6-2pro:Venus-PTS1, RcU6-3pro:Venus-PTS1, RcU6-4pro:Venus-PTS1, RcU6-5pro:Venus-PTS1, and RcU6-6pro:Venus-PTS1 constructs. The DNA of each plasmid was introduced into castor leaf cells using a gene gun [20]. The introduction of plasmid DNAs without the *U6* promoter resulted in no Venus fluorescence in peroxisomes (Figure 2A). However, the introduction of the RcU6-1pro:Venus-PTS1 plasmid resulted in Venus fluorescence in peroxisomes, which appeared as spherical spots in castor leaf cells. This indicated that *RcU6-1* promoter could transcribe *Venus-PTS1*. Similarly, the introduction of the other five *RcU6* promoters driving the *Venus-PTS1* fusion also produced fluorescence signals in peroxisomes (Figure 2C–G). The results show that the six newly cloned *RcU6* promoters are transcriptionally active in castor cells. Judging from the fluorescence pattern of Venus-PTS1, it can be concluded that there is no difference in the six *U6* promoter activities. These results also suggest that the *Venus* gene was correctly transcribed and translated, and the Venus protein was transported to peroxisomes, indicating that the *U6* promoter is functional when cloned upstream of the *Venus* gene. Thus, this study provides a simple and robust technique for verifying promoter function in plant species for which transformation techniques are not yet established.

### 3.3. Truncation of Rc U6 Promoters

**Figure 3:**
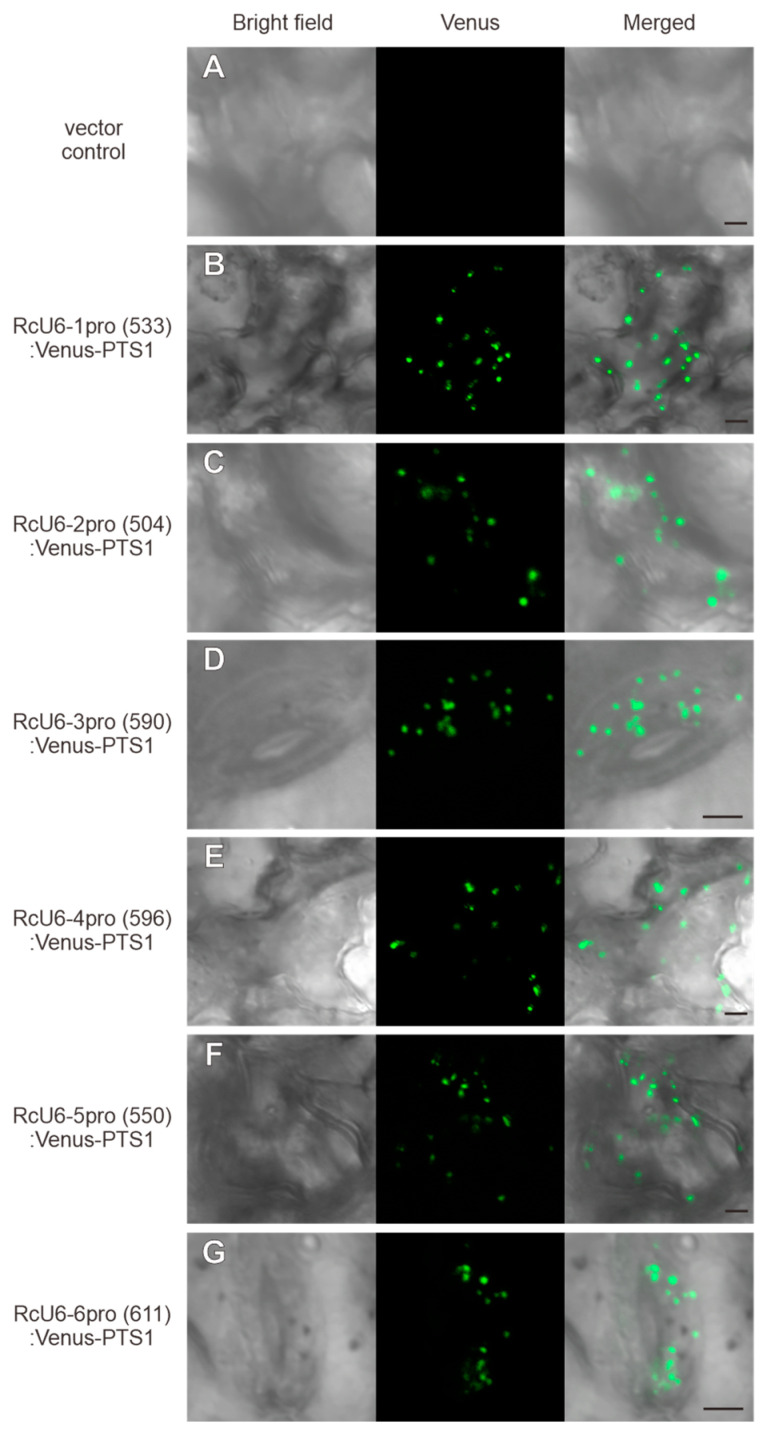
Functional verification of *RcU6* promoters truncated to approximately 500 bp. The vector without the *U6* promoter (**A**), RcU6-1pro(533):Venus-PTS1 (**B**), RcU6-2pro(504):Venus-PTS1 (**C**), RcU6-3pro(590):Venus-PTS1 (**D**), RcU6-4pro(596):Venus-PTS1 (**E**), RcU6-5pro(550):Venus-PTS1 (**F**), and RcU6-6pro(611):Venus-PTS1 (**G**) were separately introduced into castor leaf cells by particle bombardment. The numbers in brackets indicate the length of the promoter. Scale bars in the merged pictures represent 5 µm.

**Figure 4:**
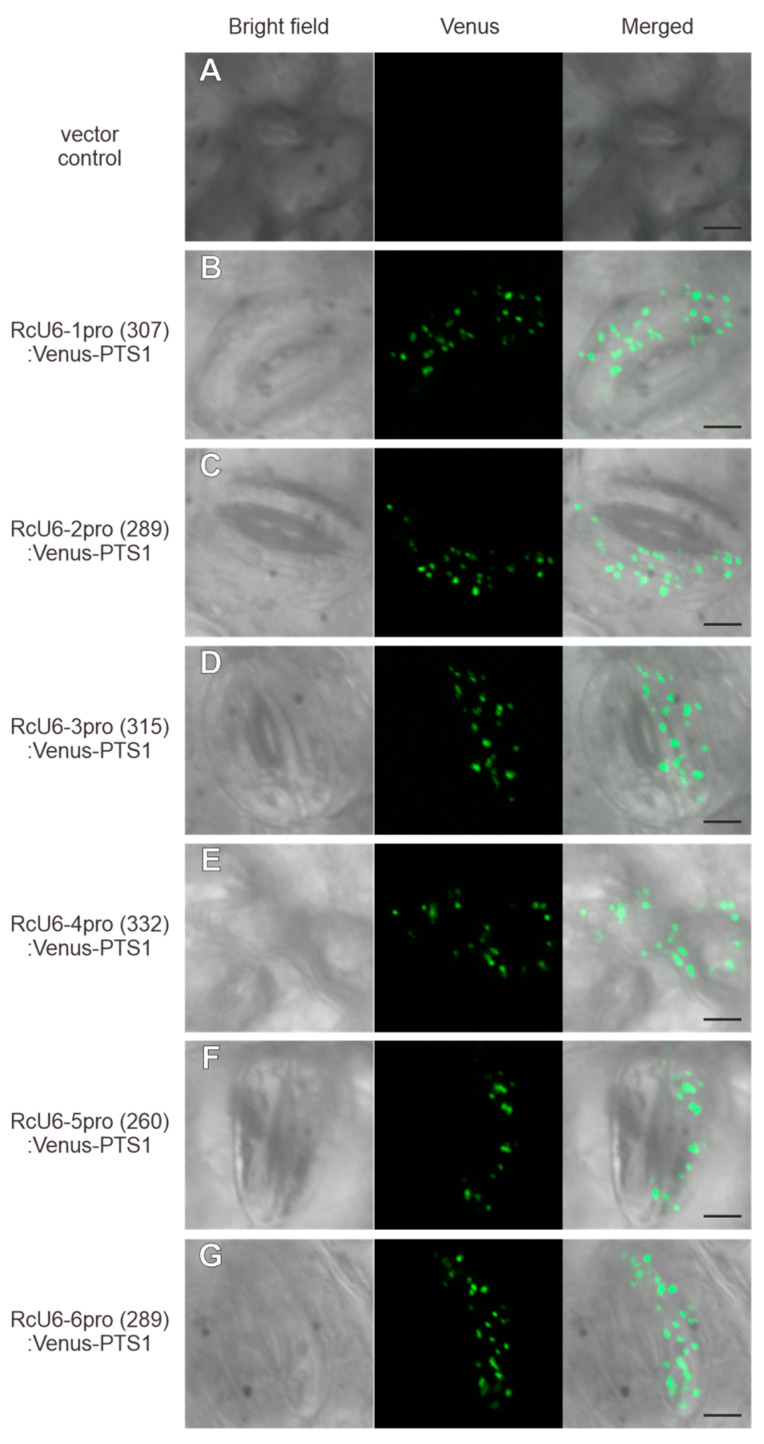
Functional verification of *RcU6* promoters truncated to approximately 300 bp. The vector without the *U6* promoter (**A**), RcU6-1pro(307):Venus-PTS1 (**B**), RcU6-2pro(289):Venus-PTS1 (**C**), RcU6-3pro(315):Venus-PTS1 (**D**), RcU6-4pro(332):Venus-PTS1 (**E**), RcU6-5pro(260):Venus-PTS1 (**F**), and RcU6-6pro (289):Venus-PTS1 (**G**) were separately introduced into castor leaf cells by particle bombardment. The numbers in brackets indicate the length of the promoter. Scale bars in the merged pictures represent 5 µm.

The ability of *U6* promoters to express *gRNA*s has been well studied. In Arabidopsis, an approximately 100 bp sequence upstream of the transcription start site of the *Rc-1* gene has been shown to function well for driving gene expression. Genome editing in plants differs from that in animals; since it is difficult to inject gRNA and Cas9 nuclease into egg cells in plants, unlike in animals, the CRISPR/Cas9 cassette is introduced into the plant genome [9,13,32]. Therefore, shortening the size of the transgene helps in increasing transformation efficiency, and the most effective way of shortening the size of the CRISPR/Cas9 cassette is by truncating the *U6* promoter. Therefore, we attempted to truncate the *RcU6* promoters to approximately 500 bp from the transcription start size. The truncated promoters, namely, *RcU6-1pro*(533), *RcU6-2pro*(504), *RcU6-3pro*(590), *RcU6-4pro*(596), *RcU6-5pro*(550), and *RcU6-6pro*(611), were cloned into the R4pGWB401 vector along with the *Venus-PTS1* fusion, and the resulting plasmids were transiently expressed in castor leaf cells by particle bombardment. The introduction of plasmid DNA without the inserted promoter resulted in no Venus fluorescence in peroxisomes (Figure 3A). However, peroxisome-localized Venus fluorescence was observed in castor leaf cells transformed with RcU6-1pro(533):Venus-PTS1, RcU6-2pro(504):Venus-PTS1, RcU6-3pro(590):Venus-PTS1, RcU6-4pro(596):Venus-PTS1, RcU6-5pro(550):Venus-PTS1, and RcU6-6pro (611):Venus-PTS1 constructs (Figure 3B–G). These results show that *U6* promoters truncated to approximately 500 bp in length are active in castor cells. In some crops, endogenous promoters truncated to approximately 300 bp have been reported to show activity [15,17,33]. Therefore, we truncated the *RcU6* promoters to approximately 300 bp from the transcription start site, and named them *RcU6-1pro*(307), *RcU6-2pro*(289), *RcU6-3pro*(315), *RcU6-4pro*(332), *RcU6-5pro*(260), and *RcU6-6pro*(289). The introduction of plasmid DNA without the inserted promoter resulted in no Venus fluorescence in peroxisomes (Figure 4A). However, fluorescence was detected in the peroxisomes of castor leaf cells transformed with RcU6-1pro(307):Venus-PTS1, RcU6-2pro(289):Venus-PTS1, RcU6-3pro(315):Venus-PTS1, RcU6-4pro(332):Venus-PTS1, RcU6-5pro(260):Venus-PTS1, and RcU6-6pro (289):Venus-PTS1 constructs (Figure 4B–G). These results demonstrate that the *RcU6* promoters, truncated to approximately 300 bp in length, are also transcriptionally active.

## 4. Conclusions

Our study demonstrates that six *RcU6* promoters truncated to approximately 300 bp are functional in castor cells. These results improve the potential of genome editing technology in castor, an important commercial crop, and provide a strategy for the construction of castor-optimized CRISPR/Cas9 cassettes in future studies. Moreover, our results show that transient expression analysis of *U6* promoters using the particle delivery method is an effective approach for the functional verification of promoters in plant species for which transformation methods have not yet been established, thus facilitating the generation of genetically modified crops.

## Figures and Tables

**Table 1:** Summary of U6 snRNA genes identified in the genome sequence of castor (ASM1957865v1) using the transcribed region of the Arabidopsis *U6-1* snRNA gene as the query sequence.

Gene Name	Chromosome No.	Region (bp)	Sequence Identity
*RcU6-1*	8	15,427,322–15,427,419	96/98 (98%)
*RcU6-2*	8	16,221,161–16,221,064	97/98 (99%)
*RcU6-3*	6	19,921,764–19,921,668	96/97 (99%)
*RcU6-4*	6	21,889,600–21,889,503	97/98 (99%)
*RcU6-5*	1	35,147,644–35,147,547	97/98 (99%)
*RcU6-6*	10	15,041,917–15,041,820	96/98 (98%)
*RcU6-7*	1	32,438,768–32,438,688	77/81 (95%)
*RcU6-8*	7	28,298,413–28,298,329	82/85 (95%)

## Data Availability

The data presented in this study are available upon request from the corresponding author.

## Supplementary Materials

The following supporting information can be downloaded at: https://www.mdpi.com/article/10.3390/genes14071327/s1, Table S1: List of primers used in this study.
