# Supplementary material for: Cloning and Functional Verification of Endogenous U6 Promoters for the Establishment of Efficient CRISPR/Cas9-Based Genome Editing in Castor (Ricinus communis)
Source: Genes (Basel). 2023 Jun 23;14(7):1327. doi: 10.3390/genes14071327 (PMC10379810; doi:10.3390/genes14071327)
Supplement: Supplementary file 1 [file genes-14-01327-s001.zip › genes-2432895-supplementary.pdf]

Table S1. Primer sequences used in this study.

Primer name	5'-sequence-3'
B4-RcU6-1pro_F	ATAGAAAAGTTGTTTATTGCTCCAGTCCAAGGTCAC
B4-RcU6-1pro(533)_F	ATAGAAAAGTTGTTTAGTTGGCAGCCTTCAGATTTC
B4-RcU6-1pro(307)_F	ATAGAAAAGTTGTTTTGCAATTATCCAGTCCTGCT
B1R-RcU6-1pro_R	TTTGTACAAACTTGCAGCATGGTGCTTGCTTGTAGG
B4-RcU6-2pro_F	ATAGAAAAGTTGTTTGCCAATAATCACATCAAACAGG
B4-RcU6-2pro(504)_F	ATAGAAAAGTTGTTTGCACAAGGCAATAGATTCAAC
B4-RcU6-2pro(289)_F	ATAGAAAAGTTGTTAAATGCAGTCAGTGAAGTTGC
B1R-RcU6-2pro_R	TTTGTACAAACTTGCACTTCAGAAGCTAGCTTGATTTTC
B4-RcU6-3pro_F	ATAGAAAAGTTGTTGTTTGGTGGGAGAATCAAGGAG
B4-RcU6-3pro(590)_F	ATAGAAAAGTTGTTTGGTTCAATAGCAGCTTTGGTG
B4-RcU6-3pro(315)_F	ATAGAAAAGTTGTTTCAGGCAGCCATGTGAAAGTT
B1R-RcU6-3pro_R	TTTGTACAAACTTGCCAACGGTGTCTGTTTGCCCTAATTC
B4-RcU6-4pro_F	ATAGAAAAGTTGTTTCCGACATAATGCTCCAGAGTC
B4-RcU6-4pro(596)_F	ATAGAAAAGTTGTTGAACCGGAAATGAGTTGACAAG
B4-RcU6-4pro(332)_F	ATAGAAAAGTTGTTATAATTAAATGGTTATTCGTTATTGTG
B1R-RcU6-4pro_R	TTTGTACAAACTTGCAGCATCATGCTGCCTCCCGTTTG
B4-RcU6-5pro_F	ATAGAAAAGTTGTTTTTCCTTCTTGGTAGGGTTTGC
B4-RcU6-5pro(550)_F	ATAGAAAAGTTGTTAATTACACATAAAATTGAATTACACATGTC
B4-RcU6-5pro(260)_F	ATAGAAAAGTTGTTTTTCTGGGAGGTATGCATCAA
B1R-RcU6-5pro_R	TTTGTACAAACTTGCAGTAGCATGTCGTTTTTGTTTACTTATAAAC
B4-RcU6-6pro_F	ATAGAAAAGTTGTTATTTCAGTTGCCGAAACTCTCC
B4-RcU6-6pro(611)_F	ATAGAAAAGTTGTTATGTTACACATGACAAGCAATACTAA
B4-RcU6-6pro(289)_F	ATAGAAAAGTTGTTCAATGTTTTCTTTTATGGAATTCTCG
B1R-RcU6-6pro_R	TTTGTACAAACTTGCCCAACCAGCTGTTTGCCCTAATTC
B1-Venus_F	AAAAAGCAGGCTTTATGGTGAGCAAGGGCGAGG
B2-VenusPTS1_R	AGAAAGCTGGGTT TCATAGCTTCGAAACCTTGTACAGCTCGTCCAT

Experiment
cloning for RcU6-1pro
cloning for RcU6-1pro(533)
cloning for RcU6-1pro(307)
cloning for RcU6-1pro, RcU6-1pro(533) and RcU6-1pro(307)
cloning for RcU6-2pro
cloning for RcU6-2pro(504)
cloning for RcU6-2pro(289)
cloning for RcU6-2pro, RcU6-2pro(504) and RcU6-2pro(289)
cloning for RcU6-3pro
cloning for RcU6-3pro(590)
cloning for RcU6-3pro(315)
cloning for RcU6-3pro, RcU6-3pro(590) and RcU6-3pro(315)
cloning for RcU6-4pro
cloning for RcU6-4pro(596)
cloning for RcU6-4pro(332)
cloning for RcU6-4pro, RcU6-4pro(596) and RcU6-4pro(332)
cloning for RcU6-5pro
cloning for RcU6-5pro(550)
cloning for RcU6-5pro(260)
cloning for RcU6-5pro, RcU6-5pro(550) and RcU6-5pro(260)
cloning for RcU6-6pro
cloning for RcU6-6pro(611)
cloning for RcU6-6pro(289)
cloning for RcU6-6pro, RcU6-6pro(611) and RcU6-6pro(289)
cloning for Venus-PTS1
